# Hemodynamic monitoring during weaning from mechanical ventilation in critically ill pediatric patients: a prospective observational study

**DOI:** 10.1186/s12887-024-05110-5

**Published:** 2024-10-26

**Authors:** Tarek Ahmed Abdelgawad, Hanan M. Ibrahim, Eman Mohamed Elsayed, Nehad Salah Abdelhamid, Somia Abdel Hamid Bawady, Ahmed R. Rezk

**Affiliations:** 1 Department of Pediatric Intensive Care Unit, Faculty of Medicine, Ain Shams University, Cairo, Egypt; 2 Department of Pediatric Cardiology Unit, Faculty of Medicine, Ain Shams University, Cairo, Egypt; 3 Department of Clinical Pathology, Faculty of Medicine, Ain Shams University, Cairo, Egypt

**Keywords:** Pediatric intensive care unit, Echocardiography, Mechanical ventilation, Weaning, Noninvasive cardiometry, Weaning failure, Pediatrics, Hemodynamic changes

## Abstract

**Background:**

Cardiovascular dysfunction is a significant factor contributing to weaning failure in mechanically ventilated children. Understanding the cardiopulmonary pathophysiological changes that occur during weaning is a prerequisite for the early recognition of weaning failure of cardiovascular origin. This study aimed to assess the effect of weaning trials on central hemodynamics and to identify the indices predictive of cardiac-related weaning failure.

**Methods:**

This prospective observational study was conducted in the Pediatric Intensive Care Unit (PICU) and included mechanically ventilated patients aged between 2 and 30 months who were on minimal ventilatory settings and ready for weaning. Patients who were hemodynamically unstable, diagnosed with neuromuscular diseases, or diagnosed with cardiac diseases were excluded. Hemodynamic parameters were evaluated during weaning from ventilation via echocardiography and noninvasive cardiometry during pressure support (PS) ventilation and at the end of the spontaneous breathing trial (SBT).

**Results:**

The study included 50 patients, comprising 30 males (60%) and 20 females (40%) with ages ranging from 2 to 30 months. Echocardiography revealed a significant increase in the cardiac index **(**CI), tricuspid annular plane systolic excursion (TAPSE), and the E/A ratio at the end of SBT. Moreover, right ventricular systolic pressure (RVSP) significantly decreased. Noninvasive cardiometry revealed a significant increase in the index of contractility (ICON) and CI at the end of SBT (p-value = 0.023 and < 0.001, respectively). Of the 12 (25%) patients who failed their first extubation trial, they exhibited a significantly lower CI and TAPSE (p values = 0.001 and 0.001, respectively).

**Conclusion:**

This study identified that weaning from mechanical ventilation in children is associated with hemodynamic changes, which can impact weaning success and reveal potential ventricular dysfunction. Bedside echocardiography was found to detect cardiac dysfunctions during weaning, and noninvasive cardiometry was considered a reliable tool that supports echocardiography for detecting changing trends in CI in PICUs. However, accurate values should be confirmed by echocardiography.

## Introduction

Mechanical ventilation is a commonly used procedure in critically ill children admitted to pediatric intensive care units (PICUs), yet it is associated with potential major side effects. Prolonged mechanical ventilation increases the risk of infections, morbidity, and mortality [1]. Understanding the cardiopulmonary pathophysiology changes during mechanical ventilation and its withdrawal is essential for early identification of weaning failure of cardiovascular origin. Weaning success depends on the ability of the cardiorespiratory system to tolerate these changes [2]. Focused critical care echocardiography (FCCE) is increasingly being performed and interpreted by trained clinicians as an essential bedside tool [3]. Noninvasive technologies such as thoracic electrical bioimpedance, thoracic bioreactance, vascular unloading technique, pulse wave transit time, and radial artery applanation tonometry are currently available for cardiac output monitoring [4]. This study aimed to assess different hemodynamic changes occurring during the weaning of the pediatric population from mechanical ventilation, and to determine cardiovascular-related predictors of weaning failure.

## Methods

### Subjects

This prospective observational study included 50 children, aged 2 to 30 months, admitted to the PICU at Ain Shams University Children’s Hospital between May 2021 and September 2022. The study population included mechanically ventilated pediatric patients ready to be weaned according to the following criteria: (i) Clinical criteria: enrolled patients were awake and responsive with an intact cough reflex; no sedation was administered at the time of examination; hemodynamically stable (i.e., capillary refill time < 3 s; age-appropriate blood pressure; heart rate with no or minimum vasopressor support); physiological and metabolic status (i.e., normal ranges of serum electrolytes and glucose, body temperature < 38 °C, hemoglobin level ≥ 8–10 g/dL), adequate oxygenation; pulsed oxygen saturation (SpO_2_ > 95%); and fair nutritional status. Additional criteria included (ii) arterial blood gases: normal pH, partial pressure of arterial oxygen (PaO_2_) > 60 mmHg, and partial pressure of arterial carbon dioxide (PaCO2) < 50 mmHg; and (iii) respiratory mechanics: fractionated inspired oxygen (FiO_2_) < 40%, PaO_2_/FiO_2_ > 200, positive end-expiratory pressure (PEEP) ≤ 5 cm H_2_O, respiratory rate (RR) < 40 breaths/minute, and tidal volume (VT) > 5 ml/kg. Patients were excluded if they were hemodynamically unstable, diagnosed with neuromuscular diseases not predicted to be weaned, or diagnosed with any cardiac disease.

## Study design and data collection

The primary objective of this study was to evaluate various hemodynamic changes that occur during the weaning process from mechanical ventilation in the pediatric population and to identify cardiovascular predictors of weaning failure. The secondary objective was to evaluate the effectiveness of noninvasive cardiometry in assessing hemodynamic changes compared to echocardiography. All enrolled patients were subjected to a full, detailed medical history and thorough clinical examination.

Regarding the weaning process, we utilized PS ventilation with settings of PEEP 5 cm H2O, PS 6–8 cm H2O, and FiO2 30–40% to provide minimal yet effective ventilatory assistance to spontaneously breathing patients. We conducted a 30-minute spontaneous breathing trial (SBT) with zero pressure support (PS), 5 cm H2O positive end-expiratory pressure (PEEP), low trigger settings, tube compensation, and FiO2 of 30–40%. The use of zero PS over T-piece SBT aims to leverage the benefits of maintaining patient-ventilator connection, ensuring adequate humidification, monitoring expiratory volume, and allowing rapid introduction of ventilation if needed [5]. All patients were extubated on nasal continuous positive airway pressure (CPAP) as per our ICU protocol. Ventilator-withdrawal failure was defined as the requirement to end SBT before completing 30 min or the need for re-intubation within 48 h of extubation. Clinical failure criteria included signs of increased respiratory muscle effort, reduced level of consciousness, uncontrollable psychomotor agitation requiring sedation, excessive sweating, and cyanosis. Additionally, failure was indicated by PaO2 levels below 50–60 mmHg, PaCO2 levels above 50 mmHg or an increase of more than 8 mmHg, SaO2 below 88–90% with FiO2 above 0.5, pH below 7.32 or a decrease of more than 0.07, respiratory rate exceeding 40 breaths/min, tachycardia or arrhythmias, high blood pressure for age, or increased blood pressure of ≥ 20% [6]. Extubation failure was defined as the need for re-intubation within 48 h of extubation.

Mechanical ventilator settings, including FiO2, peak inspiratory pressure (PIP), PEEP, mean airway pressure (MAP), RR, VT, and minute ventilation (VT × RR), were recorded for all patients before initiation and at the termination of the 30-minute SBT. All patients were subjected to the following tools before initiating and just after ending a 30-minute SBT. Transthoracic echocardiography was performed by a single observer under the supervision of a pediatric cardiologist using a 3–8 MHz pediatric cardiac transducer using the Samsung HM70A Ultrasound System to measure the left ventricular ejection fraction (LVEF), left ventricular (LV) stroke volume (SV), cardiac output, cardiac index **(**CI) was calculated, tricuspid annular plane systolic excursion (TAPSE), right ventricular (RV) and LV end-diastolic areas (EDA) to calculate the RVEDA/LVEDA ratio, maximal velocities of mitral E and A waves to calculate the E/A ratio, and right ventricular systolic pressure (RVSP). Noninvasive cardiometry was performed through electrical bioimpedance using the Cardiometer ICON^®^ to measure CI, the index of contractility (ICON), systemic vascular resistance (SVR), corrected flow time (FTc), stroke volume variation (SVV), and thoracic fluid content (TFC).

### Data analysis

The data were collected, revised, coded, and entered into the Statistical Package for Social Science (IBM SPSS) version 23. The quantitative data with a parametric distribution are presented as the mean, standard deviation, and range. Qualitative variables are presented as numbers and percentages. The comparison between groups regarding qualitative data was performed using the chi-square test and/or Fisher’s exact test when the expected count in any cell was less than 5. Comparisons between two independent groups with quantitative data and parametric distributions were performed using an independent t test, while comparisons of nonparametric data were performed using the Mann‒Whitney test. Normality was assessed using the Shapiro-Wilk test. Spearman correlation coefficients were calculated to assess the correlation between two quantitative parameters in the same group. A receiver operating characteristic (ROC) curve was used to assess the cutoff points for sensitivity, specificity, positive predictive value (PPV), negative predictive value (NPV), and area under the curve (AUC). Univariate logistic regression analysis was used to assess the odds ratio (OR) and 95% confidence interval (CI) of the most important factors associated with extubation failure. The confidence interval was set to 95%, and the margin of error accepted was set to 5%. PASS11 program for sample size calculation was used, assuming a weaning failure rate of 40%. This percentage was based on the extubation failure rate in our ICU in the year preceding study initiation. The sensitivity of echocardiology parameters for the prediction of failure was set at 80%, and specificity was set at 80%. With a sample size of 50 patients, it is possible to detect sensitivity with a power of 80% and specificity with a power of 94%, while setting the alpha error (type I error) at 0.05. Therefore, a p value > 0.05 was considered nonsignificant, a p value < 0.05 was considered significant, and a p value < 0.01 was considered highly significant.

## Results

Fifty pediatric patients were included in this study–30 males (60%) and 20 females (40%). The ages of the patients ranged between 3 and 30 months, with a median (IQR) of 21 (8–28) months. Pneumonia was the most common cause of mechanical ventilation and was recorded in 16 patients (32%). Viral sepsis and septic shock were both recorded in 16% of the patient population. Thirty-eight patients (76%) had successful extubation during the first trial; however, 12 patients (24%) did not succeed during the first trial.

The mean (± SD) heart rate significantly increased at the end of SBT (130.10 ± 12.96) compared to that during PS (120.42 ± 16.25) (p-value < 0.001). Regarding echocardiography parameters, there were significant increases in CI, TAPSE, TAPSE Z score, and E/A ratio at the end of SBT compared to those during PS (p values < 0.001, < 0.001, < 0.001, and 0.002, respectively). In addition, there was a significant decrease in RVSP with a p value < 0.001 (Table 1).

**Table 1:** Echocardiography parameters during PS and at the end of SBT

	PS (*n* = 50) (mean ± SD)	SBT (*n* = 50) (mean ± SD)	Test value	*P*-value
LVEF	60.20 ± 9.17	60.00 ± 7.56	0.227•	0.821
CI (L/m/m^2^)	4.53 ± 0.51	5.04 ± 0.59	-7.917•	0.000
TAPSE (cm)	1.72 ± 0.20	1.77 ± 0.21	-4.466•	0.000
Z-score of TAPSE*	1.4 (1–1.8)	1.65 (1.22–2.1)	-4.676‡	0.000
LV stroke volume (ml)	20.30 ± 8.41	20.33 ± 8.14	-0.031•	0.976
RVSP (mmHg)	21.48 ± 6.44	16.79 ± 5.17	7.693•	0.000
LVEDA/RVEDA	1.46 ± 0.30	1.53 ± 0.36	-1.586•	0.119
E/A	1.32 ± 0.35	1.45 ± 0.36	-3.201	0.002

• Independent t-test; ‡ Mann Whitney test

* Median (IQR)

Patients who failed extubation had lower TAPSE and TAPSE Z score during PS (p values = 0.011 and 0.039, respectively) and at the end of SBT (p values = 0.001 and 0.004, respectively) with lower CIs at the end of SBT (p value = 0.001) (Tables 2 and 3).

**Table 2:** Relationships between extubation outcomes and echocardiography parameters during PS

Echocardiography parameters	Success (*n* = 38) mean ± SD	Failed (*n* = 12) mean ± SD	Test value	*P*-value
LVEF	61.31 ± 9.85	56.67 ± 5.52	1.552•	0.127
CI (L/m/m^2^)	4.56 ± 0.45	4.43 ± 0.69	0.813	0.420
TAPSE (cm)	1.76 ± 0.17	1.60 ± 0.24	2.629•	0.011
Z-score*	1.44 (1–1.83)	1 (0.5–1.45)	-2.062‡	0.039
LV stroke volume (ml)	20.58 ± 7.75	19.42 ± 10.59	0.414•	0.681
RVSP (mmHg)	21.37 ± 6.42	21.83 ± 6.75	-0.216•	0.830
LVEDA/RVEDA	1.46 ± 0.27	1.45 ± 0.40	0.065•	0.948
E/A	1.32 ± 0.33	1.35 ± 0.44	-0.259	0.797

• Independent t-test; ‡ Mann Whitney test

* Median (IQR)

**Table 3:** Relationships between extubation results and echocardiography parameters at the end of SBT

Echocardiography parameter	Success (*n* = 38) (mean ± SD)	Failed (*n* = 12) (mean ± SD)	Test value	*P*-value
LVEF	60.87 ± 7.68	57.25 ± 6.72	1.463•	0.150
CI (L/m/m^2^)	5.19 ± 0.50	4.56 ± 0.63	3.559	0.001
TAPSE (cm)	1.82 ± 0.16	1.60 ± 0.25	3.508•	0.001
Z-score*	1.69 (1.4–2.1)	0.94 (0.52–1.55)	-2.913‡	0.004
LV stroke volume (ml)	20.41 ± 7.76	20.08 ± 9.63	0.119•	0.906
RVSP (mmHg)	16.49 ± 5.13	17.75 ± 5.40	-0.732•	0.468
LVEDA/RVEDA	1.55 ± 0.32	1.44 ± 0.47	0.919•	0.362
E/A	1.44 ± 0.34	1.49 ± 0.44	-0.480	0.634

• Independent t-test; ‡ Mann Whitney test

* Median (IQR)

**Fig. 1:**
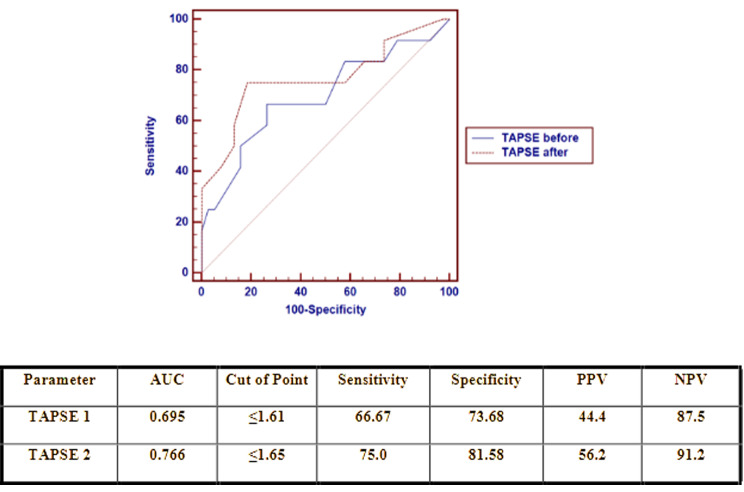
Receiver operating characteristic (ROC) curve of the TAPSE during PS and after SBT as a predictor of extubation failure

The cutoff point for TAPSE during PS for differentiating between patients who failed extubation and those who were successfully extubated was ≤ 1.61 cm, with a sensitivity of 66.67%, specificity of 73.68%, and AUC of 0.695. In comparison, the cutoff point for TAPSE at the end of SBT for differentiating between patients who failed extubation and those who were successfully extubated was ≤ 1.65 cm with a sensitivity of 75.0%, specificity of 81.58% and AUC of 0.766 (Fig. 1).

**Fig. 2:**
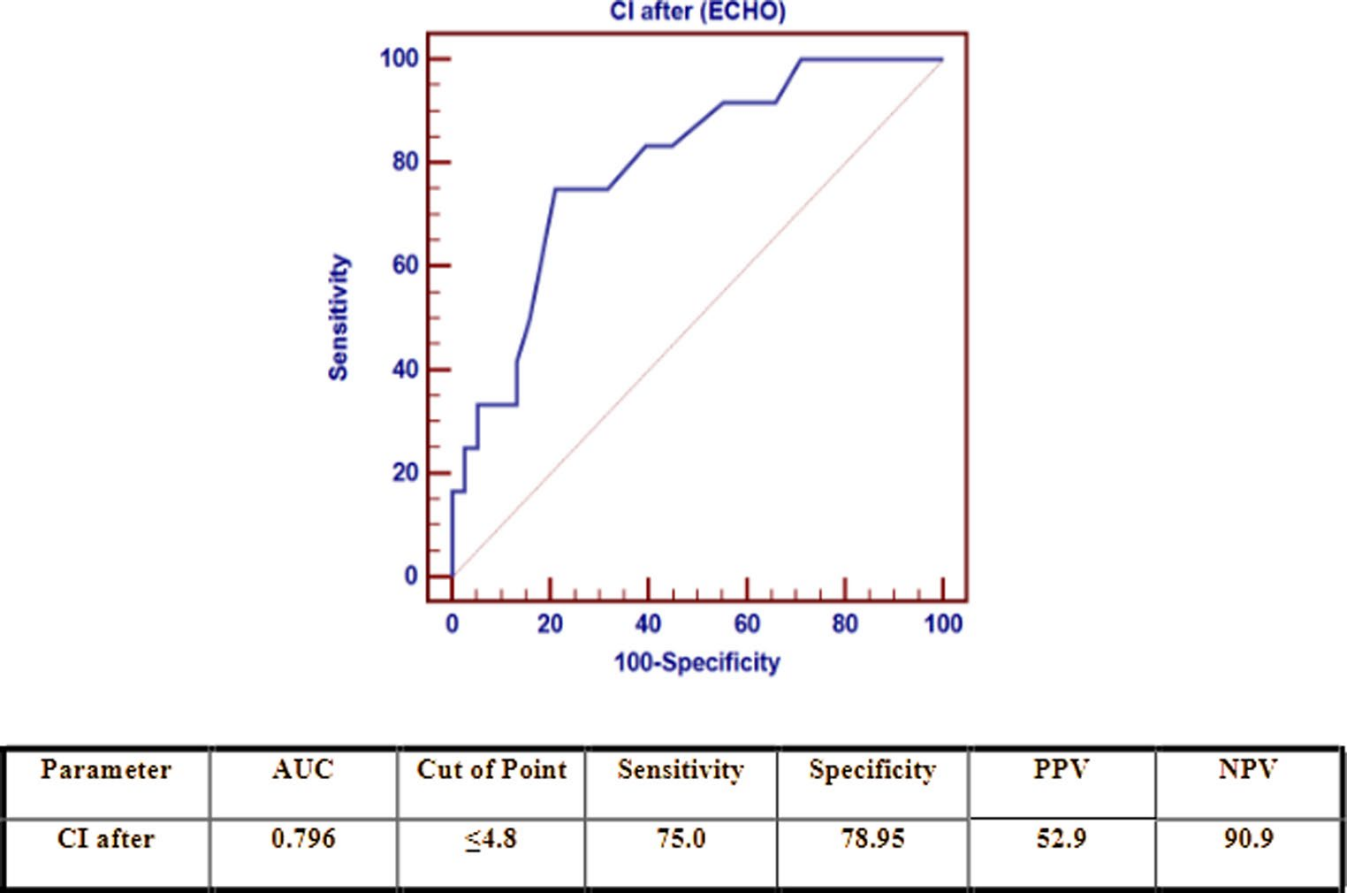
Receiver operating characteristic (ROC) curve of CI by echocardiography after SBT as a predictor of failure

The cutoff point for CI by echo at the end of SBT to distinguish between patients who were not successfully extubated was ≤ 4.8 L/m2/min, with a sensitivity of 75.0%, specificity of 78.95%, and AUC of 0.796 (Fig. 2).

Noninvasive cardiometry revealed significant increases in ICON and CI at the end of SBT compared to those during PS (p values = 0.023 and < 0.001, respectively) (Table 4).

**Table 4:** Noninvasive cardiometry parameters during PS and at the end of SBT the mean (± SD) CI values measured by noninvasive cardiometry (5.19 ± 0.72) were similar to those measured by echocardiography (5.04 ± 0.59) at the end of SBT (p-value = 0.105). However, the values measured by noninvasive cardiometry (4.89 ± 0.68) were greater than those measured by echocardiography (4.53 ± 0.51) during PS (p-value < 0.001). There was a positive correlation between CI measured by noninvasive cardiometry and echocardiography during PS and at the end of SBT (Fig. 3), and the difference was evaluated via a bland-Altman diagram (Fig. 4)

	PS (*n* = 50) (mean ± SD)	SBT (*n* = 50) (mean ± SD)	Test value•	*p*-value
ICON	83.38 ± 26.94	90.40 ± 29.87	-2.341	0.023
CI (L/m/m^2^)	4.89 ± 0.68	5.19 ± 0.72	-3.751	0.000
SVV	19.16 ± 7.75	19.24 ± 8.21	-0.102	0.919
TFC	43.34 ± 16.51	41.40 ± 18.60	0.871	0.388
FTC	268.06 ± 47.52	255.28 ± 47.65	1.958	0.056
SVR	3218.84 ± 1709.54	2992.08 ± 1146.67	1.992	0.052

•: Paired t-test

**Fig. 3:**
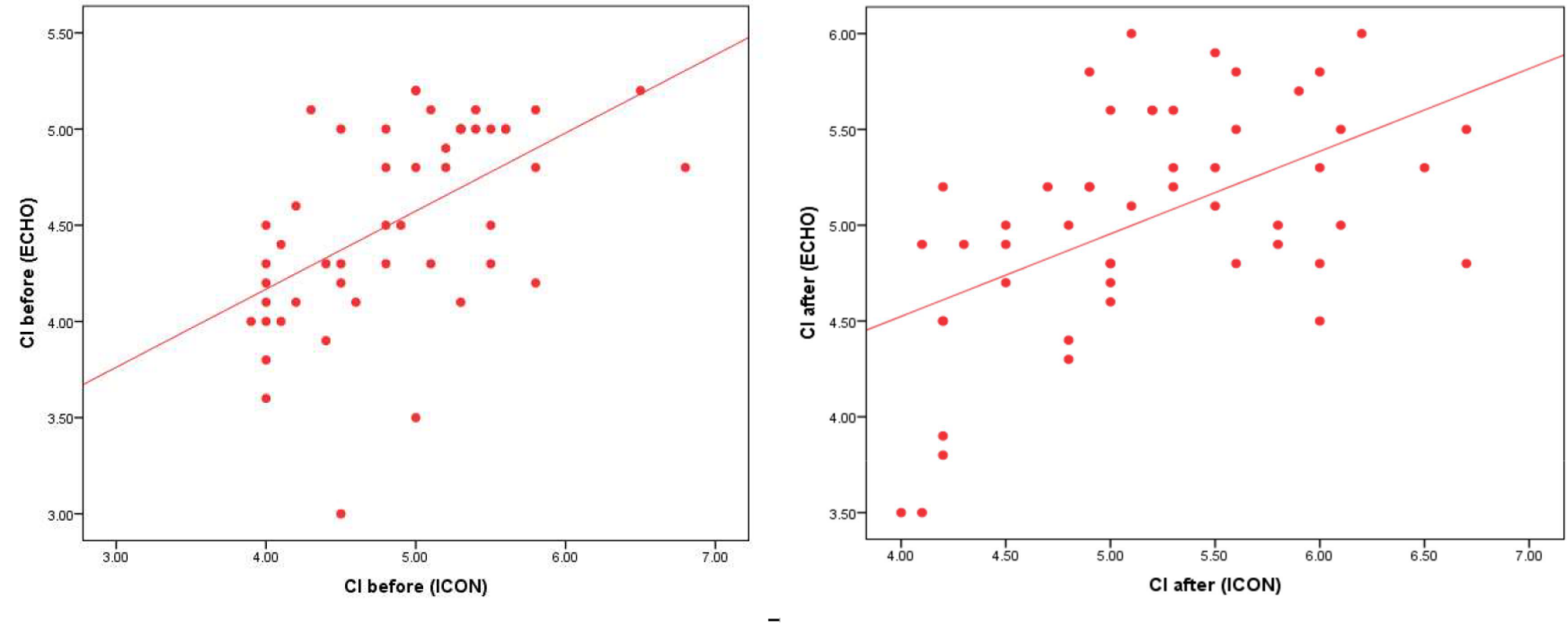
Correlations between CI by echocardiography and noninvasive cardiometry. **a** shows the correlation between CI measured via echocardiography and noninvasive cardiometry during PS. **b** shows the correlation between CI measured via echocardiography and noninvasive cardiometry after SBT

**Fig. 4:**
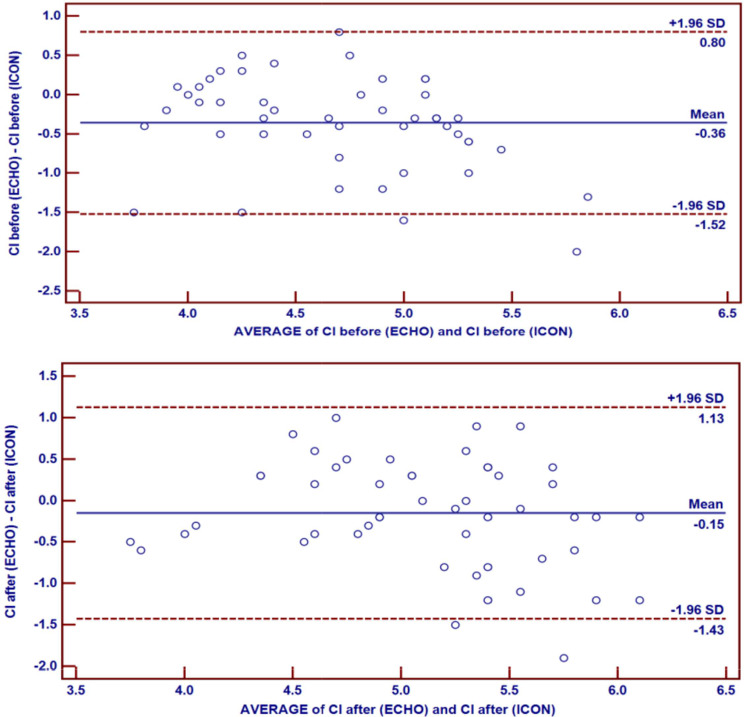
The difference between echocardiography and noninvasive cardiometry for measuring **CI.:a** Bland‒Altman plot showing the difference between echocardiography and noninvasive cardiometry measurements of CI during PS. **b**: Bland‒Altman plot showing the difference between echocardiography and noninvasive cardiometry measurements of CI after SBT

## Discussion

Cardiovascular dysfunction is a recognizable factor contributing to weaning failure, as observed by previous studies in difficult-to-wean adults on mechanical ventilation [7–9]. The utilization of echocardiography to detect cardiovascular dysfunction in the intensive care setting has grown significantly over the past two decades [10]. This study aimed to assess the different hemodynamic changes that occur during the weaning of pediatric patients from PS mechanical ventilation to SBT. Hemodynamic instability was recognized during the transition from positive-pressure mechanical ventilation to spontaneous ventilation [11]. At the end of the SBT, significant increases in mean heart rate, CI, TAPSE, TAPSE Z score, and E/A ratio were recorded, while RVSP significantly decreased. Patients who failed extubation had lower TAPSE, TAPSE Z scores, and CI, indicating them as potential predictive parameters of extubation failure. Noninvasive cardiometry showed significant increases in ICON and CI at the end of SBT. Positive correlations between CI measured by noninvasive cardiometry and echocardiography were observed.

Heart rate increased significantly at the end of SBT compared to that during PS in our study. This increase was similar to that reported in studies by *Trifi* et al. [12] and *Caille* et al. [9], who reported that the transition to T-trial increased HR significantly. The elevation in heart rate during SBT could be attributed to an increase in adrenergic tone, likely resulting from rising serum catecholamine levels during the discontinuation of positive pressure ventilation, as previously documented [13].

Several guidelines and recommendations highlighted the importance of using echocardiography in a wide spectrum of clinical settings in the ICU, including those for cardiovascular dysfunction [4, 14–16]. In this study, CI significantly increased at the end of SBT; however, there was no increase in stroke volume at the end of SBT. Therefore, we believe that this increase in CI was directly impacted by the increase in HR, which eventually influenced cardiac output. *Trifi* et al. reported that cardiac output significantly increased during SBT due to a significant increase in stroke volume [12]. However, the CI in our study was lower at the end of SBT in patients with failed weaning. *Gerbaud* et al. observed an increase in CI at the end-SBT in ventilated adult patients who were successfully weaned (3.3 [3.06–3.77] vs. 3 [2.68–3.3] L/min/m^2^, *P* < 0.001). CI remained unchanged in patients with weaning failure [17]. We suggest that the cutoff point for CI by echocardiography at the end of SBT to distinguish between failed and successful extubation cases is ≤ 4.8 L/m^2^/min, with a sensitivity of 75.00% and a specificity of 78.95%. This could provide a simple, noninvasive guidance for predicting weaning success in ventilated pediatric patients.

Diastolic dysfunction is an essential factor contributing to cardiac-related weaning failure. The E/A ratio increased at the end of SBT, suggesting an alteration in diastolic function in our study population. This alteration in diastolic dysfunction could be an impact of suggested factors, including pulmonary edema, ventricular dysfunction, myocardial ischemia, volume overload, or intrathoracic pressure variations during weaning. *Bedet* et al. [18] observed no difference in the E/A ratio in patients who were successfully weaned during the second SBT (*p* = 0.736); however, the E/A ratio significantly increased at the end of the second SBT in patients with failed weaning (*p* = 0.003). In our study, the E/A ratio in patients who achieved successful or failed weaning was similar at the end of SBT. Therefore, our study revealed that regardless of weaning outcome, the E/A ratio was not considered a predictor of weaning failure.

TAPSE significantly increased at the end of SBT, while lower TAPSE values during PS and SBT were associated with weaning failure. Similarly, *Roche-Campo* et al. [19] reported that TAPSE was also lower in patients with prolonged weaning (*p* = 0.03). Our study revealed that the cutoff point for TAPSE during PS for differentiating between failed and successful extubation was ≤ 1.61 cm, with a sensitivity of 66.67% and specificity of 73.68%. The cutoff point for TAPSE at the end of SBT for differentiating between failed and successful extubation was ≤ 1.65 cm, with a sensitivity of 75.0% and specificity of 81.58%. An adult study by *Papaioannou* et al. [18] showed that patients with prolonged weaning had decreased TAPSE compared to those with short weaning (14.59 ± 1.56 versus 19.13 ± 2.59 mm, respectively). They recorded a cutoff point of 1.64 cm, with a sensitivity of 85.00% and specificity of 75.00%, predicting a prolonged weaning process. Right ventricular systolic dysfunction in prolonged weaning patients could be related to left ventricular diastolic dysfunction, unlike what was reported in adult chronic obstructive pulmonary disease (COPD) patients by *Daif et al.* [20], in which there was no difference in TAPSE values between failed and succeeded patients. Therefore, our study suggests that TAPSE could provide a simple, noninvasive guidance for predicting weaning failure in the pediatric population.

This study also compared echocardiography to noninvasive cardiometry in the assessment of CI during weaning. The absolute CI values measured by noninvasive cardiometry were greater than those measured by echocardiography. *Sanders* et al. [21] reported that electrical cardiometry cannot replace thermodilution or transthoracic echocardiography for the measurement of absolute cardiac output. However, our study revealed a significant positive correlation between CI measured by noninvasive cardiometry and echocardiography during PS and after SBT. *Boet* et al. [22] noted that the cardiac output measured by electrical cardiometry was positively correlated with cardiac output measured by echocardiography in hemodynamically stable preterm infants. *Xu* et al. [23] also concluded that although the absolute values of cardiac output measured by electrical cardiometry and M-mode echocardiography were not interchangeable, the cardiac output distributions in both was similar. On the basis of our findings, electrical cardiometry cannot replace transthoracic echocardiography, considered the standard method for noninvasive cardiac imaging and monitoring hemodynamics. However, electrical cardiometry has proven to be an acceptable operator-independent trending tool for both cardiac output and CI assessment in pediatric patients on mechanical ventilation.

### Strengths and limitations

To our knowledge, this study is the first to assess hemodynamic changes during weaning from PS mechanical ventilation and predictors of weaning failure in the pediatric population. However, this study has some limitations that need to be accounted for. Our study did not assess heart rate variability due to the lack of measurement tools, including invasive arterial line insertion, which is not the usual practice in our PICU. Although the sample size may be considered low compared to that of adult studies, the number of patients is fairly reasonable, considering that this was a single-center observational study in a pediatric population. We believe that a larger sample size from more diverse pediatric populations will add valuable results to our findings. Although thermodilution is a more accurate method to assess cardiac output and CI, we could not use it in our study due to its invasive nature and associated complications.

## Conclusion

This study found that weaning from mechanical ventilation is associated with hemodynamic changes, which can impact weaning success. Echocardiography is considered a valuable tool that may raise suspicion of ventricular dysfunction, which can contribute to weaning failure and require further evaluation. Additionally, noninvasive electrical cardiometry was highlighted as a supportive tool for monitoring hemodynamic trends in the PICU, though it should be complemented by echocardiography for accurate absolute measurements. This approach highlights the importance of thorough cardiovascular assessment during the weaning process and addresses a critical knowledge gap in pediatric intensive care. Echocardiography should be used as part of a comprehensive evaluation, not as the sole determinant of extubation readiness. Further research with larger, more diverse populations is needed to validate these findings and determine the optimal role of echocardiography in weaning protocols.

## Data Availability

The data presented in this study are available upon request from the corresponding author. The data are not publicly available due to the use of other unpublished articles based on this database.

## Abbreviations

AUC: Area Under the Curve
CI: Cardiac Index
COPD: Chronic Obstructive Pulmonary Disease
CPAP: Continuous Positive Airway Pressure
E/A: Ratio of Early (E) to Late (A) Ventricular Filling Velocities
EDA: End-Diastolic Area
FCCE: Focused Critical Care Echocardiography
FTc: Corrected Flow Time
FiO2: Fraction of Inspired Oxygen
HR: Heart Rate
ICON: Index of Contractility
ICU: Intensive Care Unit
IQR: Interquartile Range
LVEDA: Left Ventricular End-Diastolic Area
LVEF: Left Ventricular Ejection Fraction
MAP: Mean Airway Pressure
NPV: Negative Predictive Value
OR: Odds Ratio
PEEP: Positive End-Expiratory Pressure
PICU: Pediatric Intensive Care Unit
PIP: Peak Inspiratory Pressure
PPV: Positive Predictive Value
PS: Pressure Support
PaCO2: Partial Pressure of Arterial Carbon Dioxide
PaO2: Partial Pressure of Arterial Oxygen
ROC: Receiver Operating Characteristic
RR: Respiratory Rate
RVEDA: Right Ventricular End-Diastolic Area
RVSP: Right Ventricular Systolic Pressure
SBT: Spontaneous Breathing Trial
SD: Standard Deviation
SV: Stroke Volume
SVR: Systemic Vascular Resistance
SVV: Stroke Volume Variation
SaO2: Oxygen Saturation
SpO2: Pulsed Oxygen Saturation
TAPSE: Tricuspid Annular Plane Systolic Excursion
TFC: Thoracic Fluid Content
VT: Tidal Volume
